# The Effect of Antibiotics on Associated Bacterial Community of Stored Product Mites

**DOI:** 10.1371/journal.pone.0112919

**Published:** 2014-11-11

**Authors:** Jan Kopecky, Marta Nesvorna, Marketa Mareckova-Sagova, Jan Hubert

**Affiliations:** 1 Epidemiology and Ecology of Microorganisms, Crop Research Institute, Prague, Czechia; 2 Biologically Active Substances in Crop Protection, Crop Research Institute, Prague, Czechia; Federal University of Viçosa, Brazil

## Abstract

**Background:**

Bacteria are associated with the gut, fat bodies and reproductive organs of stored product mites (Acari: Astigmata). The mites are pests due to the production of allergens. Addition of antibiotics to diets can help to characterize the association between mites and bacteria.

**Methodology and Principal Findings:**

Ampicillin, neomycin and streptomycin were added to the diets of mites and the effects on mite population growth (*Acarus siro*, *Lepidoglyphus destructor* and *Tyrophagus putrescentiae*) and associated bacterial community structure were assessed. Mites were treated by antibiotic supplementation (1 mgg^−1^ of diet) for 21 days and numbers of mites and bacterial communities were analyzed and compared to the untreated control. Bacterial quantities, determined by real-time PCR, significantly decreased in antibiotic treated specimens from 5 to 30 times in *A. siro* and *T. putrescentiae*, while no decline was observed in *L. destructor*. Streptomycin treatment eliminated *Bartonella*-like bacteria in the both *A. siro* and *T. putrescentiae* and *Cardinium* in *T. putrescentiae*. *Solitalea*-like bacteria proportion increased in the communities of neomycin and streptomycin treated *A. siro* specimens. *Kocuria* proportion increased in the bacterial communities of ampicillin and streptomycin treated *A. siro* and neomycin and streptomycin treated *L. destructor*.

**Conclusions/Significance:**

The work demonstrated the changes of mite associated bacterial community under antibiotic pressure in pests of medical importance. Pre-treatment of mites by 1 mgg^−1^ antibiotic diets improved mite fitness as indicated accelerated population growth of *A. siro* pretreated streptomycin and neomycin and *L. destructor* pretreated by neomycin. All tested antibiotics supplemented to diets caused the decrease of mite growth rate in comparison to the control diet.

## Introduction

Interactions between arthropods and microbes result in symbiotic associations, which involve both vertical and horizontal transfer mechanisms [1]. Specialized feeding on highly restricted diets and utilization of low digestible substrates (e.g. cellulose, lignin) require association with microorganisms [2], [3]. The resulting associations aid microorganisms in obtaining nitrogen, sterols, vitamins and essential amino acids [4], [5], [6], while microbial digestive enzymes support digestive systems of arthropods by interacting with their endogenous enzymes [7].

Stored product mites successfully colonize human made habitats. *Acarus siro, Lepidoglyphus destructor* and *Tyrophagus putrescentiae* feed and develop on stored plant and animal material including grain, oil seeds, dog feed, cheese, dried milk and dry ham, as well as on microscopic fungi [8]. The food sources are nutritionally unbalanced, providing high amount of some nutrients, but lacking others and for such situation the symbiosis with microorganisms is beneficial [9].

The mite associated bacteria were hypothesized to interact with their hosts in the utilization of chitin and cellulose, the most abundant structural polysaccharides in plant and fungal cell walls present in soil and man-made habitats [10], [11], [12]. The stored product mites can feed on Gram-possitive bacteria [13], however, supplementation of the rearing diet with *Micrococcus luteus* accelerated the population growth of *L. destructor* but not of *A. siro* and *T. putrescentiae* [14]. The associated bacteria enhance mite colonization of human habitats and utilization of new food sources. Except of those bacteria, the mites are attacked by pathogenic or parasitic bacteria [15]. However, associations between mites and bacteria have not yet been assessed in detail.

Antibiotics represent a tool for exploring the ecology of animal gut-associated communities [16] because they may help to understand the role of microbial communities in arthropods [9]. The host-microbe interactions were studied using antibiotics in termites [17], carabid beetles [9], hemiptera [18] and other insects. It is suggested that antibiotics cause (i) direct antibiotic toxicity (ii) decreased food intake, (iii) indirect effect on the symbiotic bacteria involved in basic physiological functions, such as food digestion, (iv) and infection resulted from an antibiotic-resistant pathogenic microorganism [19].

The work focused on demonstrating the effects of antibiotics on mites of medical importance. The selected antibiotics (ampicillin, neomycin and streptomycin) have different spectrum of target taxa but all are broad spectrum antibiotics effective on Gram-positive and negative bacteria [20]. Ampicillin is a broad-spectrum antibiotic inhibiting peptidoglycan synthesis. Streptomycin targets mainly Gram positive. Neomycin is a broad-spectrum antibiotic inhibiting translation and again [20]. The goal of the study was to evaluate changes of mite fitness after antibiotic treatments and determine effects on associated bacterial communities caused by three antibiotics with different spectra and mode of action.

## Materials and Methods

### Mites

Stored product mites *Acarus siro* L., *Tyrophagus putrescentiae* (Schrank) (Acari: Acaridae), and *Lepidoglyphus destructor* (Schrank) (Acari: Glycyphagidae) came from laboratory cultures maintained at the Crop Research Institute, Prague, The Czech Republic (CZ). Both *A. siro* and *T. putrescentiae* were collected from the grain storage inBustehrad (50°9′19″N 14°11′19″E) CZ, in 1996; *L. destructor* was collected from Food processing factory, Prague (50°9′4″N 14°31′29″E), CZ, in 1965. All species were collected by Eva Zdarkova. The stored product mites are not protected and were not collected in the protected area. The collector Eva Zdarkova had a permission to collect samples on the farm issued by the owners. The mites were mass-reared in plastic chambers derived from IWAKI tissue cell cultures with filter cap plugs (P-Lab, Praha, CZ). The chambers were kept in desiccators (P-Lab) with a saturated KCl solution providing 85% relative humidity and incubated at 25±1°C in the dark [21] Before the experiments, the mites were collected from the rearing chambers using a paint brush and a dissection Stemi 2000 C stereomicroscope (C. Zeiss, Jena, Germany).

### Rearing and experimental diets

The rearing diet contained a mixture of powdered oat flakes, wheat germ and Pangamin, a dried yeast biomass (Rapeto, Bezdružice, CZ) (10/10/1 w/w/w). The rearing diet (control) was powdered by blender, sieved at 0.5 mm sieves and sterilized by heating to 70°C for 0.5 h. The diet of 50±5 mg was placed to every IWAKI chamber. The antibiotics, ampicillin sodium salt (cat No. A0166, Sigma-Aldrich, Saint Louis, USA), neomycin trisulfate salt hydrate (cat No. N6386, Sigma-Aldrich) and streptomycin sulfate salt (cat No. S9137, Sigma-Aldrich) were added to the rearing diets, separately. The treatments included antibiotic concentrations of 0.1, 1, 10, and 30 mg of antibiotics per g of diet (mgg^−1^). The control was the rearing diet without supplementation.

### Effect of antibiotic supplement on mite growth

Fifty unsexed adults from a 21 days old culture were added to each chamber. Ten replicates of each species, type of antibiotic and concentration were set. The chambers were incubated in desiccators at the same conditions as describe above for 21 days The experiment was terminated by addition of 10 mL of 80% ethanol per chamber. Mites were counted under the dissection stereomicroscope.

### Effects of antibiotic pre-treatment on mites

Fifty unsexed adults from a 21 days old culture were added to each chamber on 100±10 mg of 1 mg g^−1^ antibiotic containing diets. The concentration was selected based on the previous experiments to ensure that there is a suppressive effect but the mites’ populations are still able to grow. The antibiotics ampicillin, neomycin and streptomycin diets were added separately. The control had no antibiotics. Ten replicates per antibiotic type and species of mites were set. The rearing conditions were the same as described above. After 21 days 10 adults were removed by brush from every chamber and placed to a new chamber with 50±5 mg of un-supplemented (control) rearing diet. The chambers were maintained in the same rearing conditions as in experiment assessing the population growth for 21 days. The experiment was terminated by addition of 10 mL of 80% ethanol to every chamber. Mites were counted under the dissection stereomicroscope.

### Data analyses for biotests

For antibiotic treatment, the final population numbers (N) showed normal distribution and were analyzed by analysis of covariance (ANCOVA). Final population numbers were dependent, and type of antibiotics, concentration, mite species and their interactions were independent variables. The ANCOVA models showed higher R^2^ values when the antibiotic concentration was transformed according to the formula LN(concentration +1.10^−7^) than the models without transformation. Finally, the interaction of final population number and antibiotic concentration were analyzed separately per antibiotic type and mite species using regression models. Effective doses of antibiotic concentration reducing final population to 50% (EC_50_) in the comparison to control were estimated from the model with 95% confidence intervals.

To evaluate the effect of antibiotic pre-treatment, final population numbers (N) were analyzed using analysis of variance (ANOVA) separately for each mite species. In the model, the dependent variable was the final population number and the factors were the pre-treatments (control, ampicillin, neomycin and streptomycin). Dunnet two-side test was applied to indicate the difference of the pre-treatment from the control.

The analyses were done in XLSTAT 2007 (Addinsoft USA, New York, NY, USA) and QC-Expert (TriloByte Statistical Software, s.r.o., Pardubice, CZ).

### The samples of mites for PCR and plating

1,000±100 mites were reared in IWAKI chambers on 250±50 mg diets containing 1 mg g-1 of antibiotic as was describe above. Three replicates per antibiotic and mite species were set. The control diet contained no antibiotic. The mites in IWAKI chambers were cultivated under conditions as in experiment describing the effect of antibiotics on population growth. The experiment was terminated by collection of mites. The mites (10±1 mg) were removed by paint brush from plugs or inner surfaces of the chambers using the dissection stereomicroscope.

The mites were weighed using Mettler AE 240 microbalance (Mettler-Toledo, Columbus, OH, USA), with accuracy of 10 µg in Eppendorf tubes. Two tubes per one chamber were analyzed. Each tube was filled by 1 mL of bleach for 1 minute, the sample was centrifuged at 3,000 g (1 minute) and the bleach was replaced by 1 mL of absolute ethanol for 1 minute and centrifuges again 3,000 g (1 minute). The ethanol was replaced by PBST (phosphate buffered saline with the detergent Tween 20, 3.2 mM Na_2_HPO_4_, 0.5 mM KH_2_PO_4_, 1.3 mM KCl, 135 mM NaCl, 0.05% Tween 20).

The sample was cleaned 3 times with PBST using centrifugation. Then, the mites were homogenized in 100 µl of PBST by 2 mL glass homogenizer with Teflon-pestle (Kavalier, Sázava, CZ). The first set of tubes was used for total DNA extraction. DNA was extracted using Dneasy Tissue kit (Qiagen, Valencia, CA, USA), and cleaned with GeneClean Turbo kit (MP Biomedicals, Solon, USA).

The second sets of tubes contain the mite homogenates was used for plating. The homogenates were diluted in PBST and plated on Tryptic Soy Broth agar plates (HiMedia, Mumbai, India). The plates were incubated for 7 days at 25±0.5°C and colony forming units were checked. The colonies of different morphologies were identified using 16 S rRNA gene amplifications and sequencing [22].

### PCR

PCR amplification of 16S rRNA gene was performed with universal bacterial primers – UF: 5′-AGA GTT TGA TYM TGGC- 3′ (position 8–23 according to *E. coli*) and UR: 5′-GYT ACC TTG TTA CGA CTT-3′ (position 1496–1514) [23]. Amplification was performed using C1000 Thermal Cycler (Bio-Rad, Hercules, USA). PCR reaction mixture contained in 25 µl total volume: 200 µM dNTPs, 3 mM MgCl_2_, forward and reverse primers (100 nM each), 0.5 unit *Taq* polymerase (all Promega, Madison, WI, USA) and 300 ng template DNA (i.e. mite genomic DNA with bacterial DNA). The amplification conditions were as follows: 2 min at 94°C, and 30 cycles of 90 s at 94°C, 90 s at 50°C, and 60 s at 72°C, followed by final extension for 10 min at 72°C and 4°C hold [23].

Resulting PCR products were purified with Wizard SV Gel and PCR Cleanup Kit (Promega). The PCR products from bacterial primers were cloned using pGEM-T Easy Vector (Promega) and sequenced by the Sanger dideoxy method (Macrogen, Seoul, Korea). Three independent samples per treatment (control, ampicillin, neomycin and streptomycin) and mite species were prepared and pooled for the subsequent analysis.

### 16S RNA gene library

Nearly full-length sequences of 16S rRNA gene were assembled with CodonCode Aligner, version 1.5.2 (CodonCode Corporation, Dedham, MA, USA) and assigned to bacterial taxa using Ribosomal database project naïve Bayesian rRNA classifier [24]. The sequences representing bacterial communities associated with *A. siro* (233 clones), *L. destructor* (189) and *T. putrescentiae* (280) were obtained. As a cultivable part of the respective bacterial communities, 177 isolates from *A. siro* (54 strains), *L. destructor* (68) and *T. putrescentiae* (55) were identified. The cloned sequences were deposited in GenBank under Accession Nos. JX064540–JX064766 and KM464000–KM464486, and the sequences from isolates under Accession Nos. JX064767–JX064911. Separately for clones and isolates, the 16S rRNA gene libraries were classified to operational taxonomic units (OTU_97_) using Mothur v. 1.32 program [25].

### Quantitative PCR

The previously prepared DNA samples were used in three technical replicates per each sample. 5′-ACTCCTACGGGAGGCAGCAG-3′ and Eub518a: 5′-ATTACCGCGGCTGCTGG-3′. According to the previously published protocol [26], nearly full-length 16S RNA gene amplicon of Streptomyces sp. was used as a standard. The amplification was done on a StepOnePlus Real-Time PCR System (Life Technologies, Carlsbad, CA, USA) using 96-well plates with GoTaq qPCR Master Mix (Promega) containing SYBR Green as a double-stranded DNA binding dye.) The amplification consisted of 40 cycles including denaturation (30 s at 95°C), annealing (35 s at 54°C), and elongation (45 s at 72°C). The inhibition was tested by serial DNA dilution from each site. Melting curves were recorded to ensure qPCR specificity [26]. Baseline and threshold calculations were performed with the StepOnePLus software. To evaluate the effect of antibiotic treatment, the obtained numbers of copies were analyzed using the analysis of variance (ANOVA) separately for each mite species. Dunnet two-side test was applied to indicate the difference of the treatment from the control.

## Results

### The suppressive effect of antibiotics on growth rate of mites

All diets supplemented with antibiotics decreased the growth rate of *A. siro*, *L. destructor* and *T. putrescentiae* (Table 1). The ANCOVA model (*F_(22,427)_ = 104; P<0.0001*) showed that the final population was significantly influenced by antibiotic concentration (*F = 104; P<0.0001*)) tested species (*F = 33; P<0.0001*), and type of antibiotic (*F = 27; P<0.0001*). The significant effect was observed for the interaction of species×type of antibiotic (*F = 29; P<0.0001*). Other interactions were not significant.

**Table 1:** The final population density (N) of species of mites on antibiotic additive diets and fitted concentration EC_50_ is an effective concentration of antibiotic s in diets for 50% population growth reduction in the comparison to growth on control diet.

Species	Antibiotics (mgg^−1^)	Observed data (N)					Regression model				
		0	0.1	1	10	30	R^2^	a	b	c	Fit EC_50_ mgg^−1^
*Acarus siro*	Ampicillin	1183	728	484	275	200	0.95	−2.95	−89	513	0.37
		(71)	(111)	(25)	(47)	(56)		(−3.7/−2.2)	(−100/−78)	(483 /543)	(0.20/0.55)
	Neomycin	1179	1059	501	391	304	0.87	−5	−115	689	2.23
		(74)	(89)	(73)	(24)	(57)		(−7/−4)	(−133/−96)	(640/739)	(1.11/3.67)
	Streptomycin	1280	1118	883	452	292	0.94	−7	−142	847	4.95
		(102)	(97)	(49)	(71)	(41)		(−8 /−6)	(−155/129)	(811/882)	(3.52/7.03
*Lepidoglyphus destructor*	Ampicillin	1718	1398	1144	603	185	0.87	−10	−202	1060	2.66
		(108)	(208)	(129)	(216)	(57)		(−12/−8)	(−231/−173)	(982/1139)	(1.51/7.03)
	Neomycin	1685	414	338	197	116	0.98	2	−56	304	0.0004
		(105)	(60)	(60)	(40)	(26)		(1/3)	(−66/−45)	(275/332)	(0.0001/0.001)
	Streptomycin	1685	1465	1106	428	231	0.93	−11	−214	1050	2.38
		(96)	(218)	(161)	(68)	(51)		(−12/−9)	(−236/−191)	(990/1110)	(1.7/3.07)
*Tyrophagus putrescentiae*	Ampicillin	1164	415	297	156	131	0.97	0.1	−51	293	0.003
		(95)	(36)	(18)	(20)	(18)		(−0.4/0.6)	(−60/−44)	(272/314)	(0.001/0.01)
	Neomycin	1271	751	639	290	139	0.95	−4	−108	569	0.76
		(75)	(103)	(63)	(36)	(37)		(−5/−3)	(−120/−96)	(537/602)	(0.41/1.21)
	Streptomycin	1193	1111	411	355	148	0.85	−7	−140	661	1.58
		(98)	(144)	(48)	(41)	(16)		(−8/−5)	(−164/−118)	(599/724)	(0.74/2.66)

Legend: The final population density is expressed as mean ± standard deviation; the polynomial regression (r = ax2+bx+c; x = antibiotic concentration mgg−1 of diet) describing the suppressive effect of antibiotics to growth rate of tested mites. The antibiotic concentration was transformed logarithmically (LN concentration +1×10^−7^). The fitted parameters are showed with 95% confidence limits in parenthesis.

Polynomial regressions showed the effect of antibiotics on suppression of the growth rate separately for each type of antibiotic and species (Table 1). *L. destructor* was the most sensitive to antibiotic treatment, followed by *T. putrescentiae* and *A. siro* according to EC_50_ values. Neomycin was the most suppressive to population growth rate, followed by streptomycin and ampicillin.

### The effect of antibiotic pre-treatment on mite growth

**Figure 1:**
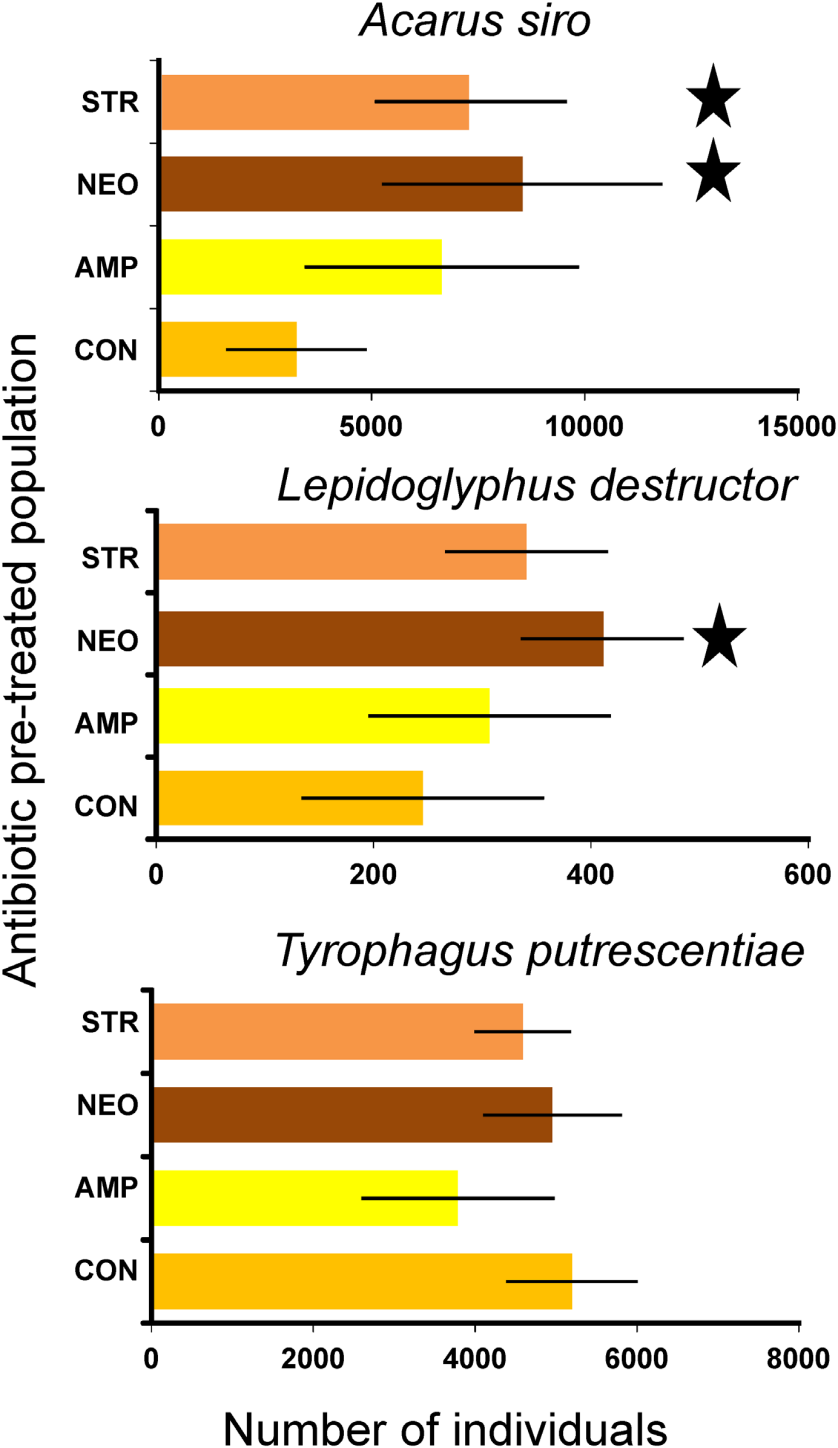
The effect of antibiotics pre-treatment on the final population size after 21 days of growth on untreated diet, averages with respective standard deviations. The Dunnet significant differences from the control are marked by asterisk; CON - untreated control, AMP –ampicillin, NEO – neomycin, STR – streptomycin.

Pre-treatment of mites with diets containing antibiotics showed no effect on *T. putrescentiae*, (ANOVA: *F_(3,20)_ = 3; P = 0.061*), but significant effects were observed for *A. siro* and *L. destructor* (ANOVA: *F_(3,20)_ = 4; P = 0.017* and *F_(3,20)_ = 3; P = 0.042*, respectively) (Figure 1). The response of population growth was similar for both species; the highest population number was observed after neomycin pre-treatment, followed by streptomycin and ampicillin, the lowest population density was in the control (see Figure 1 for significant differences).

### Bacterial communities in stored product mites

**Figure 2:**
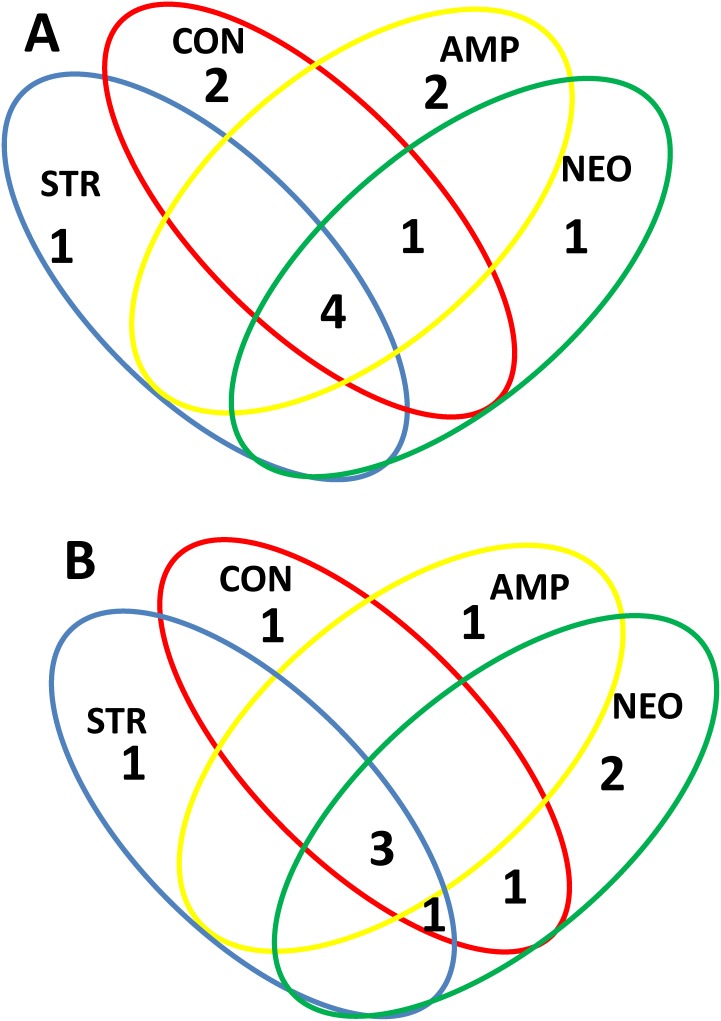
Venn’s diagram comparing the sequences in 16S rRNA library of the clones (A) and isolates (B) from mite on control and antibiotic treated diets.

Numbers of 16S rRNA gene copies quantified by qPCR showed differences between mite species decreasing from *A. siro* (2×10^5^) to *T. putrescentiae* (1×10^5^) and *L. destructor* (5×10^4^) (Figure 2). The 16S rRNA libraries from all species formed 11 OTUs_97_ of cloned sequences (Table 2) and 10 OTUs_97_ from isolated bacteria (Table 3). In *A. siro*, *Bartonella*-like bacteria and *Solitalea*-like bacteria OTUs_97_ were prevailing OTUs. *Bartonelia*-like bacteria together with *Cardinium* sp. were also prevailing OTU_97_ in T. *putrescentiae*. While *Bacillus* OTU_97_ was prevailing in *L.destructor* (Table 2). The 16S rRNA library of sequences from isolates were characterized by high a proportion of *Staphylococcus nepalenesis* OTU_97_ and *Bacillus* sp OTU_97_ in all species of mites (Table 3). In *L destructor* next 4 OTUs_97_ appeared with lower dominance then previously mentioned OTUs_97_.

**Table 2:** The analyses of 16S rRNA libraries from the clones from stored product mites reared on control diets.

OTU_97_	*A. siro*				*L. destructor*				*T. putrescentiae*				Total				GenBank match		
	C	A	N	S	C	A	N	S	C	A	N	S	C	A	N	S	I(%)	taxon	Ac. No.
1	2	7	1		50	60	14	31	15	44	47	47	67	111	62	78	99	*Bacillus cereus*	KF150374
2	38	26	15						38	17	15	1	76	43	30	1	99	*Bartonella*-like	JN236499
3	6	6	40	46									6	6	40	46	99	*Solitalea*-like	JN236468
4	1	28	2	12	3	1	17	12	3	1	2	3	7	30	21	27	99	*Kocuria* sp.	AB480758.1
5									12	20	6		12	20	6	0	99	*Cardinium* sp.	JX001272
6									7				7	0	0	0	94	*Sodalis* sp.	CP006569
7								1				1	0	0	0	2	99	*Staphylococcus* sp.	GU451172.1
8									1				1	0	0	0	95	*Virgibacillus halotolerans*	NR_108860
9											1		0	0	1	0	99	*Leuconostoc citreum*	FJ716698
10		1											0	1	0	0	97	*Paenibacillus xylanilyticus*	HQ258920
11		1											0	1	0	0	99	*Sphingomonas yabuuchiae*	NR_028634

The data from the three samples per treatment are presented altogether.

Legend: Accession number of match in GENBANK for the most similar sequences of identified bacteria I (%) describes the similarity; A– ampicillin treated diet, C – control diet, N – neomycin treated diet, S – streptomycin treated diet (all 1 mgg−1 of diet).

**Table 3:** The analyses of 16S rRNA libraries from isolates from stored product mites reared on antibiotic treated and control diets.

OTU_97_	*A. siro*				*T. putrescentiae*				*L. destructor*				Total				GenBank match		
	C	A	N	S	C	A	N	S	C	A	N	S	C	A	N	S	I(%)	taxon	Ac. No.
1	15	6	8	8	8	3	9	5	8	4	5	5	31	13	22	18	99	*Bacillus sp.*	KF788243.1
2	11	5	1		11	8	4	3	14	7	3	3	36	20	8	6	99	*Staphylococcus* *nepalensis*	AB697719.1
3									1	2	4	1	1	2	4	1	100	*Bacillus pumilus*	KJ722435.1
4								1	2		2	3	2		2	4	100	*Kocuria* sp.	KC502897.1
5							1		1				1		1		99	*Micrococcus* sp.	F465977.1
6												1	0			1	99	*Bacillus* sp.	KJ184936
7									1				1				99	*Oceanobacillus* sp.	EU709018.1
8							1						0		1		99	*Lysinibacillus* sp.	KJ191429.1
9							1						0		1		99	*Acinetobacter lwoffii*	KF993657.1
10										1			0	1			99	*Bacillus megaterium*	KJ461522.1

Legend: Accession number of match in GENBANK for the most similar sequences of identified bacteria I (%) describes the similarity; A- ampicillin treated diet, C – control diet, N – neomycin treated diet, S – streptomycin treated diet (all 1 mgg^−1^ of diet).

In the cloned library, 5 OTUs_97_ were common in the control and antibiotic treatments (Figure 2A), while 6 OTUs_97_ were specific for the treatments. In the library of isolates, 3 OTUs_97_ were common in the control and antibiotic treatments, while (Figure 2B). The antibiotic treatments has 7 unique OTUs_97_.

**Figure 3:**
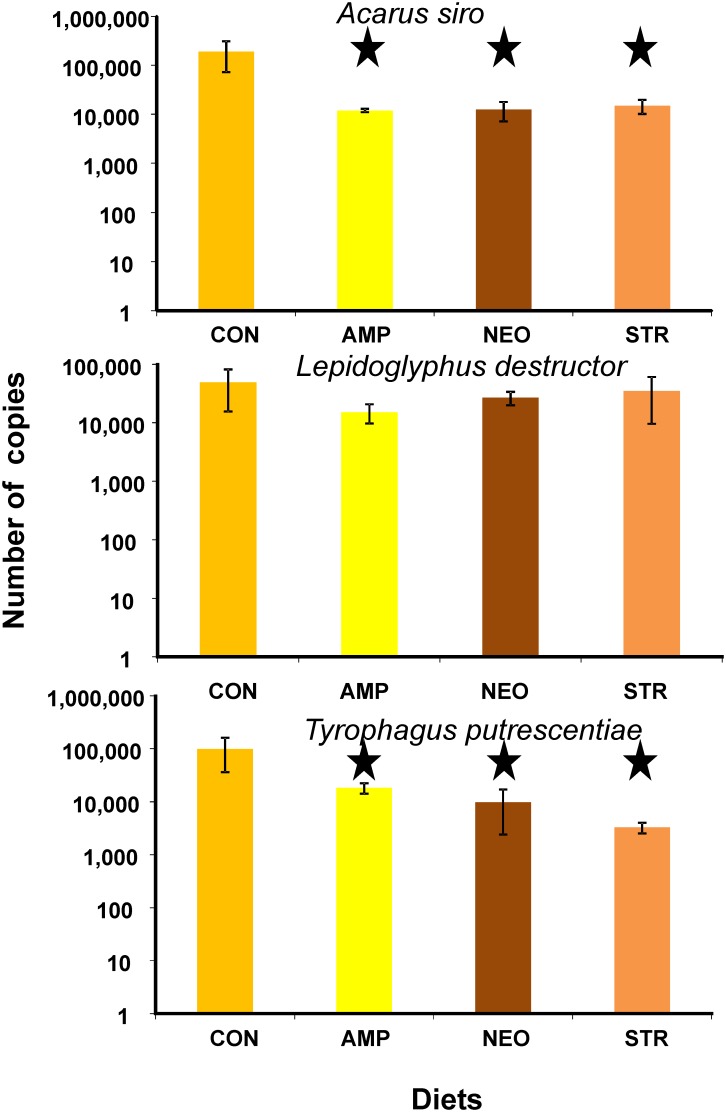
Quantitative real-time PCR of total bacteria from the DNA extracted from stored product mites reared on control and antibiotic treated diets. The numbers of copies were recalculated per one specimen, averages with respective standard deviations. The Dunnet significant differences from the control are marked by asterisk; CON - untreated control, AMP –ampicillin, NEO – neomycin, STR – streptomycin.

Antibiotics differed in their effect on bacterial community further supporting their species specific effects on mites. In *A. siro,* bacterial numbers decreased 13 to 16 times on antibiotic treated diets in comparison to the control (Figure 3). The effect was significant (*F_(3,42)_ = 22; and P<0.001*) and all antibiotic treatments differed from the control. Similar high decrease 5 to 30 times on antibiotic treated diet in comparison to the control was observed in *T. putrescentiae.* The effect was significant (*F_(3,32)_ = 10; P<0.001*) and all antibiotic treatments differed from the control. No decrease was (*F_(3,32)_ = 1.7; P = 0.18*) observed on *L. destructor* (Figure 3).

**Figure 4:**
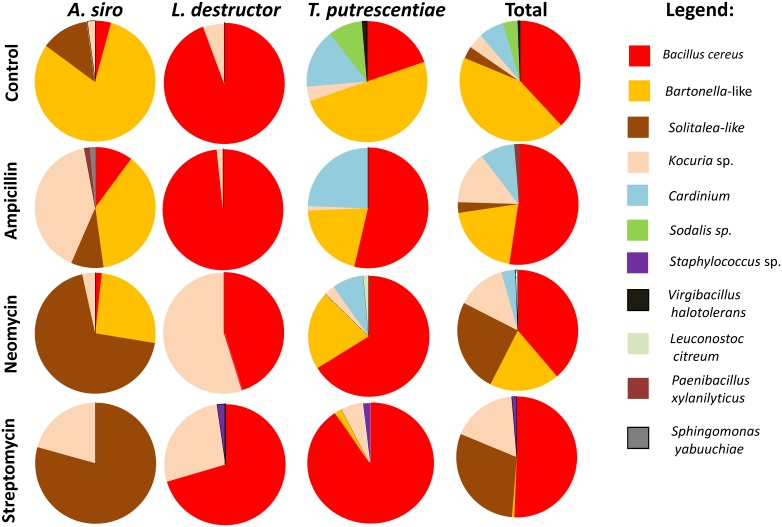
The comparison of cloned 16S rRNA sequences of stored product mites (*Acarus siro*, *Lepidoglyphus destructor* and *Tyrophagus putrescentiae*) on control and antibiotic supplementation (1 mg g^−1^ of diet) diets.

**Figure 5:**
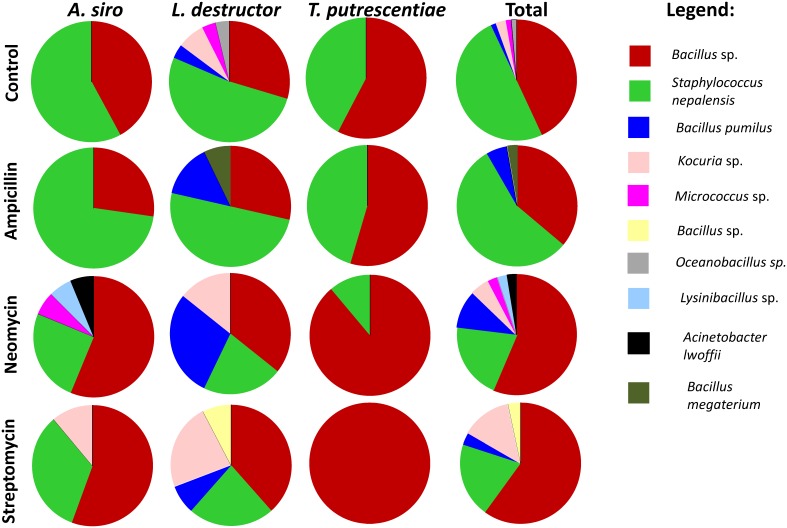
The comparison of 16S rRNA sequences from isolated bacteria from of stored product mites (*Acarus siro*, *Lepidoglyphus destructor* and *Tyrophagus putrescentiae*) on control and antibiotic supplementation (1 mg g^−1^ of diet) diets.

In the clone library of *A. siro*, *Bartonella*-like OTU_97_ proportion decreased in ampicillin and neomycin treatment and was eliminated completely with streptomycin treatment (Figure 4). *Kocuria* OTU_97_ increased in ampicillin and streptomycin treatments. *Solitalea*-like OTU_97_ predominated in neomycin and streptomycin treatments (Figure 4). The isolate library did not differ between the control and ampicillin treatment (Figure 5). The proportion of *Bacillus* OTU_97_ was higher than *Staphylococcus nepalensis* OTU_97_ in neomycin and streptomycin treatments.

In the clone library of *L. destructor*, the bacterial community after ampicillin treatment was similar to the control. The proportion of *Kocuria* OTU_97_ increased and Bacillus spp OTU_97_ decreased in neomycin and streptomycin treatments. The isolate libraries showed small difference between control and ampicillin treatments. While in neomycin and streptomycin treated specimens the proportion *Staphylococcus nepalensis* and *Bacillus* spp OTUs_97_ increased. *Bacillus pumilis* OTU_97_ and *Kocuria* spp OTU_97_ proportions increased on neomycin and streptomycin, respectively (Figure 5).

In the clone library *T. putrescentiae*, *Bartonella*-like OTU_97_ proportion decreased after all antibiotic treatments, while the proportion of *Bacillus cereus* OTU_97_ increased (Figure 4). *Cardinium* OTU_97_ was reduced on streptomycin and partly on neomycin. The isolate libraries showed small difference between the control and ampicillin treatment. The proportion of *Bacillus* OTU_97_ to the proportion of *Staphylococcus nepalensis* OTU_97_ significantly increased in neomycin treatment, while *Bacillus* sp. OTU_97_ formed exclusively the library in streptomycin treatment (Figure 5)._._

## Discussion

The size of bacterial communities of stored product mites treated by selected antibiotics decreased in all studied mite species confirming the negative effect of ingested antibiotics on the community of mite associated bacteria. The selected broad spectrum antibiotics have a different spectrum of target taxa [20], and consequently, the decrease in bacterial numbers was specific to mite species, i.e. differed by the taxonomic composition of associated bacterial communities. That might also result from a different gene pool of resistance. The antibiotic effect was suppressive to the dominant species and shifted the community to newly established species leading to the disruption of symbiotic interaction [17]. Some of the detrimental effect of the antibiotic treatment can be a result of eliminating beneficial bacteria. The decrease of some predominant bacterial taxa means an increase of proportion of bacteria resistant to the particular antibiotic [9].

Generally in our experiments, the reduction of population growth seemed to be caused by antibiotic toxicity. All tested antibiotics showed negative effect on mite growth (*A. siro*, *T. putrescentiae,* and *L. destructor*) confirming the previous results [27], [28]. Effective antibiotic doses (EC_50_) for mite suppression were rather low (up to 3 mg g^−1^) in comparison with other compounds, which were tested in feeding biotests. For example, plant volatiles, (*2E*)-hexenal, (2*E*, 6*Z*)-nonadienal and (*2E*)-nonenal had EC_50_ in range from 4 to 35 mg g^−1^ [29].

In this study, the pre-treatments of diets by neomycin and streptomycin positively affected the growth of *A. siro* and neomycin that of *L. destructor.* The bacterial communities of *A. siro* on neomycin and streptomycin treated diets were characterized by low numbers of *Bartonella*-like bacteria. The speculation is that *Bartonella*-like bacteria have an antagonistic effect to *A. siro*. In *L. destructor,* the neomycin treatment strongly decreased the proportion of *Bacillus* in the bacterial community. The *Bacillus* bacteria may have both positive and negative effects. The acaropathogenic *Bacillus thuringiensis* var. *tenebrionis* suppressed population growth of *L. destructor* in laboratory experiments [30] and *Bacillus sphaericus* caused higher mortality and prolonged development of *Dermatophagoides pteronyssinus* [31]. Other *Bacillus* species can interact with nutrient utilization or serve as food sources in mites [13], [14]. The stored product mites (e.g. *A. siro*, *Aleuroglyphus ovatus*, *Carpoglyphus lactis*, *L. destructor* and *T. putrescentiae*) are used in mass production of mite predators in the system of arthropod pest biological control in the grain stores (*Cheyletus eruditus*, *C. malaccenis*) [32], [33] or greenhouses (e.g. *Neoseiulus cucumeris*, *N. barkeri*, *N. californicus* and *Amblyseius swirskii*) [34], [35]. For such production the “good fitness” of prey mites is necessary to reach high predator numbers. The positive effect of antibiotic pre-treatment can improve the fitness of the prey mites resulting in higher population growth, and consequently in improved the predatory mite production. However the use of antibiotics for these reasons remains questionable due to the risk of spreading resistant bacterial strains.

The observed bacterial communities in analyzed stored product mites (*A. siro*, *L. destructor* and *T. putrescentiae*) reared on the control diet corresponded to the previous descriptions in these mite species [22], [36], [37], [38]. Bacterial communities of *A. siro* and *T. putrescentiae* containing symbiotic/parasitic bacteria (*Bartonella*-like, *Cardinium* and *Solitalea*-like) were 10 times more abundant than in *L. destructor*, whose bacterial community was formed mainly by *Bacillus* spp. Our previous observation of the bacterial community of *T. putrescentiae* feeding on different *Fusarium* spp. fungi indicated a possible association to some members of *Bacillus* [36]. However, both findings were at a population, not individual level, so high variability of outcomes at individual level may change our conclusions.

Bacterial communities of the three mite species differed from the bacterial community described in another stored product mite *Rhizoglyphus robin* [12]. The bacterial communities can be different due to feeding on a different diet, also [39]. The bacterial community of *T. putrescentiae* feeding on various fungal strains is well documented showing switches in bacterial community to chitinolytic strains *Pseudomonas stutzeri*, *Brevundimonas vesicularis*, *Stenotrophomonas maltophilia*, *Serratia liquefaciens* and *Serratia marcescens* [11].

The specific effects of various antibiotics on internal bacterial communities were demonstrated previously. *In* *vitro* cultivation test of cells with *Cytophaga*-like symbionts (i.e. *Cardinium*) showed that ampicillin was highly effective compared to streptomycin [40]. In the *Buchnera*-*Serratia* and aphid symbiotic system, the ampicillin treatment eliminated *Serratia* in a dose-dependent manner, while rifampicin treatment eliminated *Buchnera* in dose-independent manner [41]. Here, the treatment of mites resulted in decrease of Gram-negative *Bartonell*a-like bacteria by all antibiotics. Consequently, all used antibiotics can be applied to reduce or eliminate *Bartonella*-like species, which might be useful since the genus *Bartonella* has been suggested as horizontally transferred group in the ticks [42]. The *Cardinium* in *T. putrescentiae* was sensitive to neomycin and streptomycin, but not ampicillin. *Cardinium* belongs to the known arthropod symbionts transmitted vertically, and it can be eliminated from mites by antibiotics or heat treatment [43], [44], [45], [46].

The last group of bacteria suppressed by antibiotics was *Bacillus cereus* and similar taxa in *L. destructor*. However, *Bacillus-cereus*-like sequences formed the same OTU_97_ with those in *T. putrescentiae*, where the proportion increased. A closer identification of *Bacillus* using 16S rRNA gene is limited [47], so different *Bacillus* species could inhabit T. *putrescentiae* and *L. destructor* mites.

The decrease of above mentioned taxa lead to the proportional increase (not absolute) of taxa such as *Solitalea*-like bacteria and *Kocuria*. *Solitalea*-like bacteria were not influenced by antibiotic treatment, therefore their proportion increased in the communities of neomycin and streptomycin treated *A. siro*. Our suggestion is that *Kocuria* originates from the diet of mites, since *Kocuria* were also reported from the insects [2], [48] and related species of house dust mites [49].

In conclusion, the treatment of stored product mite by broad-spectrum antibiotics decreased the bacterial numbers in tested mites. All tested antibiotics suppressed the *Bartonella*-like bacteria, and neomycin and streptomycin suppressed *Cardinium*. This findings should be useful in production of mites for medical diagnostics (purified natural allergens) and immunomodulations [50], [51]. However, *Solitalea*-like associated group was not influenced by treatment with the antibiotics used here. In *A. siro* and *L. destructor,* the antibiotic pre-treatment (streptomycin and neomycin) can improve fitness levels, which is beneficial for mass production.

## Acknowledgments

The authors are obligated to anonymous referee for valuable comments, to Jessica Guy for comments and improving of language and Tomas Erban for antibiotic diet preparation.
